# Comparison of Oogenesis and Sex Steroid Profiles between Twice and Once Annually Spawning of Rainbow Trout Females (*Oncorhynchus mykiss*)

**DOI:** 10.1100/2012/986590

**Published:** 2012-11-19

**Authors:** Francisco Estay, Nelson Colihueque, Cristian Araneda

**Affiliations:** ^1^Piscícola Huililco Ltda., Camino Caburgua km17, Casilla 71, Pucón, Chile; ^2^Departamento de Ciencias Biológicas y Biodiversidad, Universidad de Los Lagos, Avenida Alcalde Fuchslocher 1305, Casilla 933, Osorno, Chile; ^3^Departamento de Producción Animal, Facultad de Ciencias Agronómicas, Universidad de Chile, Avenida Santa Rosa 11315, Casilla 1004, Santiago, Chile

## Abstract

This study compares the gonadosomatic index (GSI), oocyte growth (OG), gonadal histology, and plasma level concentrations of sex hormones (estradiol-17**β** (E2) and vitellogenin (V)) of twice-spawning (T-SP) and once-spawning (O-SP) females of rainbow trout throughout the additional and the normal reproductive cycle, respectively. In T-SP, the GSI values rapidly increase from May to November, in contrast to O-SP, which showed low and constant GSI values (1.19 to 14.5 and 1.19 to 0.63, resp.). T-SP exhibited a marked increase of OG in the same period, reaching a maximum diameter of 4,900 ± 141.42 **μ**m, in contrast to O-SP, which presented a slow OG. The gonadal histology of T-SP agreed with the general pattern of ovogenesis observed for O-SP (vitellogenesis, ovulation, and recrudescence); however, this process was nonsynchronous between the two breeder groups. Plasma steroid levels showed significant variation during oogenesis, which agreed with the GSI, OG, and gonadal histology patterns. The level of E2 increased to a maximum value of 26.2 ng/mL and 36.0 ng/mL in O-SP and T-SP, respectively, one or two months before the spawning event where vitellogenesis was fully active. The V concentrations followed a pattern similar to those of E2.

## 1. Introduction

Biannual spawning behavior is an atypical reproductive phenomenon exhibited by some rainbow trout broodstocks (called twice spawners), and it is characterized by two spawning events in the same year. The first spawning occurs during a normal reproductive cycle, and the second spawning occurs during an additional reproductive cycle approximately 6 months later [1–3]. The short interval between these two spawnings is an uncommon period for gonadal maturation in this species. In ordinary breeders with a single spawning event each year (once spawners), this long-term process begins a year before ovulation, and this event is critical to yield the appropriate quality and quantity of mature oocytes for reproduction [4, 5]. 

 Reproductive performance [6–10] and endocrine profile [11] studies have indicated that no major reproductive physiological disruptions occur in twice spawners, but the absence of data on gonadal maturation and oogenesis has prevented analysis of the effects of biannual spawning behavior at this level. 

 To assess whether the progress of gonadal maturation and oogenesis is affected by biannual spawning behavior, we examined both gonadal mass and histology throughout the maturation season in twice spawners, and we compared these results to those obtained from once spawners. To form a more comprehensive view of this effect, we also used analysis of the endocrine profiles of two sex hormones involved in the growth of ovaries.

 This work presents the results of the gonadal histology observations during oogenesis, the gonadosomatic index variations, and the endocrine profiles of sex hormones of twice-spawning and once-spawning rainbow trout females. These data will allow us to better understand the reproductive physiology of biannual spawners and may contribute to the development of new rainbow trout broodstocks with high fecundity.

## 2. Materials and Methods

### 2.1. Fish Used in Analysis

**Figure 1:**
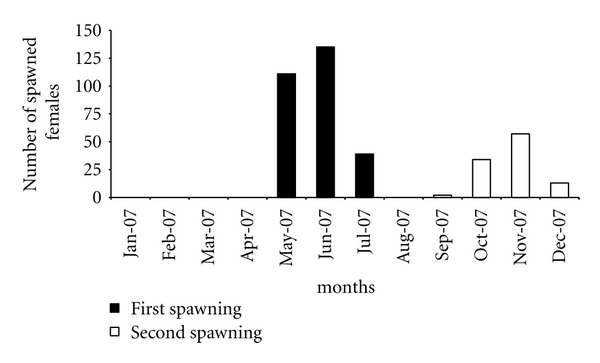
Spawning frequency of a biannual spawning female broodstock (Wt-03) of rainbow trout from Chile.

The twice-spawning population was obtained from the Piscícola Huililco Ltda rainbow trout fish hatchery located in Pucón, Chile (39°14′29.5′′S 71°50′09.8′′W). These trout originated in 2003 and belong to the Wytheville strain (Wt-03). They exhibit primary spawning during the fall (May–July) and secondary spawning during the spring (September–December), although only 37.2% of the females underwent an additional spawning (Figure 1) in this study. Once spawners from the Wt-03 broodstock (fall spawning) were used as a control with which to contrast the observations. All trout were kept in raceway-type ponds supplied with spring water at temperatures of 9 to 11°C.

### 2.2. Sample Collection

The study began immediately after the studied populations presented its normal autumn spawning in May 2007, and it finished in January 2008, one month after the twice spawners underwent their additional spawning. Samples were collected every two months during the last week of the month. Four female specimens were sacrificed each time. The ovaries were dissected for histological analysis, and a blood sample from the caudal vein was obtained for hormone determinations. 

### 2.3. Estimation of the Gonadosomatic Index

The weights of the specimens and the ovaries were determined using digital balances sensitive to 1 g (Bonzo) and 10 mg (Sartorius), respectively. The gonadosomatic index (GSI) was calculated as a percentage of the gonadal weight (Wg) over the total body weight (Wt) using the following formula: GSI = ((Wg/Wt) × 100). 

### 2.4. Histological Examinations of Ovaries and Oocyte Growth

The ovaries were fixed in a solution made up of absolute ethanol (850 mL), 40% formaldehyde (100 mL), and glacial acetic acid (50 mL) [12]. Gilson fixative (1 : 1 : 1 absolute ethanol: glacial acetic acid: chloroform and sublimate mercuric chloride to saturation) [13] was used for the fixation of oocytes with large vitelli. Ovary sections were cut to a 5 *μ*m thickness on a microtome, extended and adhered to a slide, and then they were stained with hematoxylin and eosin stain as well as Mason's trichrome stain. The Bromage and Cumaranatunga criteria [14] were used to classify the different stages of oogenesis. The oocytes' growth rates were determined by diameter measurements made on histological slides using a stereoscopic magnifier fit with a micrometer-graduated eyepiece. Five larger diameter oocytes from three slides of each female were chosen for this measurement.

### 2.5. Plasma Level Determinations of Sex Steroids

To determine the sex steroid profiles, blood samples were obtained in heparinized tubes and kept for 12 hours at 8–12°C. They were then centrifuged at 3,000 rpm for 5 min to obtain the plasma fraction, which was frozen at −80°C until hormone determination. Plasma concentration levels were analyzed for estradiol-17*β* (E2) and vitellogenin (V). The level of E2 was determined using radioimmunoanalysis (RIA) (BioSource Europe, S.A.), in which a fixed quantity of steroid labeled with ^125^I competes against the steroid present in the sample for the limited number of antibodies immobilized on the polystyrene tube wall. A standard curve was plotted, and the steroid concentration of the sample was determined by interpolation of the concentration from this standard curve. The minimum detectable quantity of E2 was 0.05 ng/mL with less than 1% cross-reactivity to other hormones. Vitellogenin levels were determined by an indirect enzyme-linked inmunosorbent assay (ELISA) method where a known “capture” antibody was first attached to the bottom of the well, the sample was added, and then a secondary (labeled) antibody was added to bind to the sample antigen. Finally, a substrate that would generate detectable signals (which correlate positively with antigen concentrations) was added. 

### 2.6. Statistical Analyses

Significant differences between means of the reproductive and physiological variables were assessed with a two-way analysis of variance (ANOVA), followed by Fisher's least significant difference (LSD) test, which allowed post hoc pairwise comparisons of the means [15]. 

## 3. Results

### 3.1. GSI and Oocyte Growth

**Figure 2:**
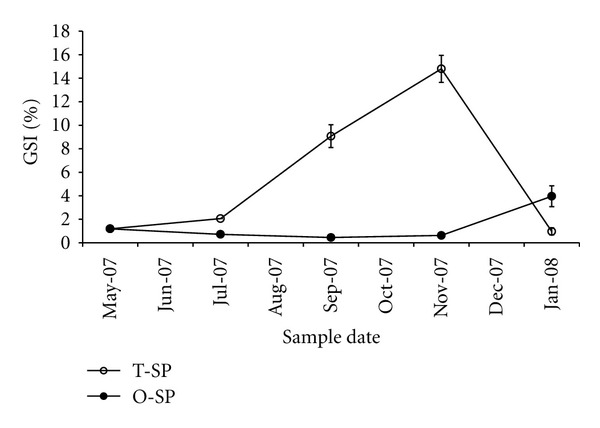
Change of the gonadosomatic index (GSI) of rainbow trout females through their gonadal reproduction cycle. O-SP: once spawners; T-SP: twice spawners (mean ± SE, *n* = 4).

The GSI values (Figure 2) of twice spawners increased from 1.19 in May to 14.5 in November, and then decreased to 1 in January, which is a pattern associated with the ovulation process for the additional reproductive cycle. In contrast, once spawners showed a low and constant GSI value whose slight increase in January is associated with the start of vitellogenesis in the normal reproductive cycle, which will finish in autumn with the annual spawning event. 

**Figure 3:**
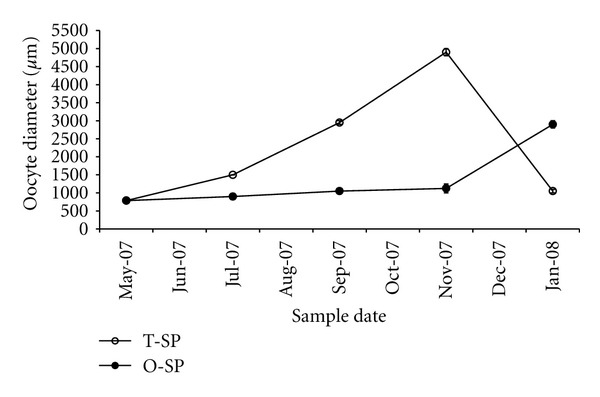
Oocyte diameter during rainbow trout oogenesis. O-SP: once spawners; T-SP: twice spawners (mean ± SE, *n* = 4).

 The oocyte growth (Figure 3) of twice spawners increased markedly from May onward, after the normal reproductive cycle of autumn, and it reached a maximum diameter of 4,900 ± 141.42 *μ*m in November when ovulation began. In contrast, once spawners presented low oocyte growth, only exhibiting an increase above 2,000 *μ*m in January when vitellogenesis began. The observed trend in oocyte growth was similar to the pattern observed for GSI.

### 3.2. Histology of the Ovary

**Figure 4:**
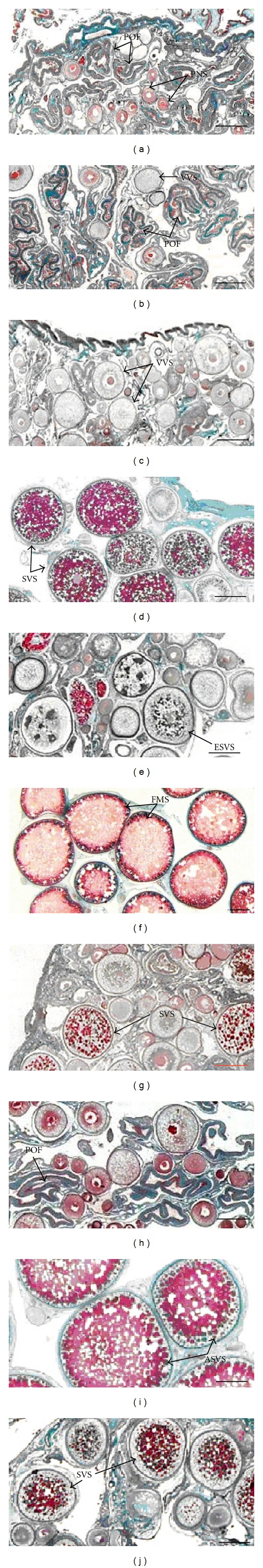
Histological sections of ovaries at different stages of development during oogenesis. (a), (c), (e), (g), (i): once spawners; (b), (d), (f), (h), (j): twice spawners. (a), (b): May; (c), (d): July; (e), (f): September; (g), (h): November; (i), (j): January. POF: postovulatory follicles; PNS: perinucleolar stage; VVS: vitellin vesicles stage; SVS: secondary vitellogenesis stage; ESVS: early secondary vitellogenesis stage; FMS: final maturation stage; ASVS: advanced secondary vitellogenesis stage. Bar scale represents 1,000 *μ*m.

In May, both once spawners and twice spawners exhibited ovary stromas that were largely composed of postovulatory follicles (Figures 4(a) and 4(b)). Some previtellogenic oocytes at the perinucleolar stage were also present along with oocytes at the vitellin vesicles stage with diameters of approximately 700 *μ*m. Overall, the gonadal histology characteristics between both classes of breeders were highly similar at this time point. 

 In July, once spawners exhibited ovary stroma composed of previtellogenic oocytes at the perinucleolar stage, along with a large population of oocytes at the vitellin vesicles stage with diameters ranging from 750 *μ*m to 800 *μ*m. These oocytes were in the primary growth stage, indicating an incipient start to the reproductive cycle (Figure 4(c)). In contrast, the majority of the oocytes in twice spawners were in full secondary vitellogenesis, with a mean diameter of 1,500 *μ*m, which reveals a high rate of vitellogenesis (Figure 4(d)). The gonadal histology characteristics between both classes of breeders were markedly different at this time point.

 In September, once spawners exhibited oocyte populations that were slowly advancing into secondary vitellogenesis, as indicated by vitellin vesicles located in the ooplasmatic region near the germinal vesicle (Figure 4(e)), and oocytes had reached a mean diameter of 1,000 *μ*m. In contrast, the oocytes in twice spawners, which had finished secondary vitellogenesis, reached a mean diameter of 3,000 *μ*m. These oocytes presented vitellin globules occupying over 90% of the ooplasm and had started the final maturation phase. Final maturation is characterized by the progressive fusion of vitellin globules along with the migration of the germinal vesicle to the periphery (Figure 4(f)). Due to these oocyte characteristics, it is expected that the twice spawners would spawn in one to two months. 

 In November, once spawners exhibited an oocyte population that continued to slowly advance into secondary vitellogenesis; the most advanced oocytes presented a vitellus deposit in a large area of the ovoplasm and reached a mean diameter of 1,100 *μ*m (Figure 4(g)). The twice spawners exhibited a clear postovulatory ovary (Figure 4(h)) whose characteristics were similar to those observed in May at the beginning of the study. The ovaries contained several postovulatory follicles and previtellogenic oocytes with a diameter of 850 *μ*m, a larger size than that observed in May. 

 In January, once spawners had finished secondary vitellogenesis, and the ovoplasm was in the vitellin globules fusion stage (Figure 4(i)). In these breeders, the oocytes had reached a mean diameter of 3,000 *μ*m, suggesting imminent ovulation. In contrast, the ovaries of twice spawners presented a very different histological morphology; the oocyte population was in active secondary vitellogenesis and reached diameters between 1,000 and 1,200 *μ*m (Figure 4(j)). These characteristics indicate rapid progress toward ovarian recrudescence. 

### 3.3. Sex Steroid Profiles

**Figure 5:**
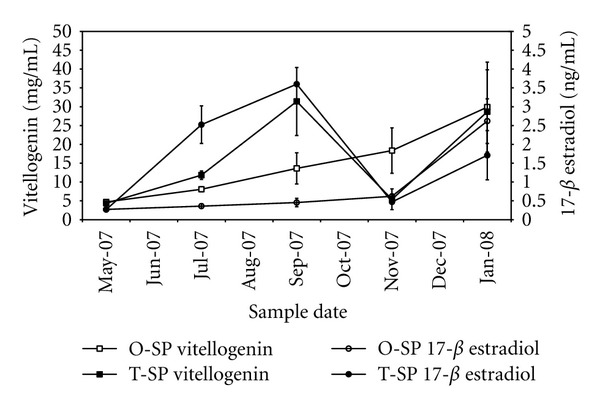
Sex steroid profiles in rainbow trout during the reproductive cycle. O-SP: once spawners; T-SP: twice spawners (Mean ± SE, *n* = 4).

Plasma steroid levels showed significant variation during the oogenesis of the different classes of breeders, which are in agreement with the data from GSI, gonadal histology and oocyte growth patterns. In once spawners, E2 concentrations gradually increased, reaching 26.2 ± 5.9 ng/mL (mean ± standard error) in January when vitellogenesis was close to its maximum activity before the normal spawning event in autumn (Figure 5). In contrast, twice spawners exhibit a marked increase in concentrations of E2 when vitellogenesis finished before the second spawning event, from 2.7 ± 0.3 in May to a maximum of 36.0 ± 0.0 ng/mL in September. The difference from the mean recorded in May was significant (*P* < 0.05). The level then decreased significantly to 4.7 ± 2.0 ng/mL in November (*P* < 0.05) during the spawning period and again showed a significant increase to 17.1 ± 6.5 in January (*P* < 0.05) prior to the first spawning event of the next year.

Vitellogenin concentrations followed a similar pattern as that observed for E2. In once spawners, this phospholipoprotein complex displayed a gradually increasing concentration throughout the breeding season, reaching a value of 29.0 ± 11.95 mg/mL in January. In contrast, twice spawners exhibited significantly increased concentrations of vitellogenin (*P* < 0.05), from 4.35 ± 0.66 mg/mL in May and 11.83 ± 1.11 mg/mL in July to a maximum of 31.40 ± 9.03 mg/mL in September, and the mean concentration decreased significantly to 5.40 ± 0.88 mg/mL in November (*P* < 0.05) when vitellogenesis finished. However, the levels of this hormone began to increase again in January and reached a value of 28.68 ± 11.14 mg/mL, a significant difference from the mean recorded in November (*P* < 0.05). This increase was likely in anticipation of a new spawning event in the autumn.

## 4. Discussion

### 4.1. Variations in GSI

Although the final GSI values for the once spawners were not recorded in this study, the maximum average GSI value observed for the twice spawners (14.8 ± 0.63) is within the range described by Aida et al. [7] for females of the same class during their additional reproductive cycle (11.1 ± 0.6 and 17.7 ± 0.5). In normal females, low GSI values have been observed at the start of ovogenesis between February and August (0.32–0.95) [16], and then a rapid increase begins in September (2.16–2.45) due to the advance of the exogenous vitellogenesis, until the GSI values peak between November and January (14.6–20.15), a period that corresponds to the month in which ovulation occurs. Therefore, the maximum GSI values observed in the twice spawners is within the lower limit of the range reported for normal rainbow trout females during ovulation. The marked increase in the GSI values of twice spawners starting in September, which is clearly a reflection of the active exogenous vitellogenesis in their ovaries, contrasts with the basal values that are observed in the once spawners during the same period. Later in January the opposite trend is seen, and it can be observed on one side by the postovulation period of the twice spawners, and on the other, by the start of vitellogenesis in the once spawners. In other words, the dynamics in both types of females are functionally coherent but seasonally nonsynchronous.

### 4.2. Oocyte Growth

The marked speed of early oocyte growth in the twice spawners indicates the commencement of a new reproductive cycle soon after the first spawning of a normal reproductive cycle. In fact, these oocytes nearly doubled their diameter every 60 days in order to reach their final size at the time of spawning in November. This pattern of oocyte growth contrasts with that of once spawners, where oocyte growth in the same period showed only a slight increase. On the other hand, all of the oocytes measured in our study (between 400 and 3,000 *μ*m) were within stages 4, 5, and 6 of the exogenous vitellogenesis period described by Van Den Hurk and Peute [16], although the twice spawners measured in November exhibited a larger oocyte (4,900 *μ*m) than could be classified in these authors' stage 7, corresponding to the oocytes' maturation period.

### 4.3. Histology of the Ovary

The histological observations of the twice spawner ovaries studied were consistent with the general pattern proposed by Van Den Hurk and Peute [16]. These authors classify rainbow trout oogenesis into three main developmental stages: (1) ovulation and previtellogenesis, (2) exogenous vitellogenesis, and (3) maturation of the oocytes. In the females we studied, the ovulation events that occurred in May and November were revealed by postovulatory follicles that could be observed in the ovarian stroma. Moreover, comparisons of ovary histology between once and twice spawners revealed the nonsynchronous dynamic of progress toward ovogenesis. Thus, whereas the twice spawners showed a rapid advance in the accumulation of vitellin globules in the ovoplasm and their consequent coalescence in large vitellin platelets in winter and spring, the once spawners showed slow and gradual progress during the same process that finishes in the summer. All the histological observations show a pattern that is consistent with the advances observed in both the GSI and the oocyte growth. These results clearly reflect the nonsynchronous processes of ovogenesis that occur in the once and twice spawners.

### 4.4. Sex Steroid Profiles

The minimum plasmatic E2 values observed postspawning in once spawners and twice spawners were similar; in both cases they reached values between 3 and 4 ng/mL. Later, these values increased to reach a peak in each class's corresponding period of maximum vitellogenic activity (26.2 and 36.0 ng/mL for the once spawners and twice spawners, resp.) one or two months before the spawning event that occurs in January for the former and in September for the latter. Even though we did not record data beyond January for the once spawners, we expect that this value would increase slightly in February because the spawning period of these females occurs between April and May. Scott et al. [17] observed that the plasmatic E2 in the normal reproductive cycle of the rainbow trout presented a higher peak of approximately 50 ng/mL, which does not fully agree with our data (31 ng/mL), although our result is similar to that reported by Schulz [18]. However, the hormone profile of E2, represented by a peak about a month before the ovulation period and a decrease to basal levels around ovulation, is similar in both types of females. In addition, our results agree with the plasmatic E2 levels observed in twice-spawning rainbow trout from Japan [18]; these females also showed a quick elevation in their E2 levels after the normal winter spawning. Interestingly, these authors also observed an E2 peak approximately one month before the additional spawning (a process that occurs during the summer in these twice spawners), with levels ranging from 30 to 45 ng/mL. 

 The variations observed in the V plasmatic levels showed a pattern that was highly similar to E2, which is in accord with the previously reported positive correlation between these hormones in the plasma during the vitellogenic growth of rainbow trout ovaries [19]. In this species, the normal cycle of maturation leads to increasing levels of vitellogenin from the beginning of spring (0.5 mg/mL) to a peak in autumn just before spawning (50 mg/mL) [5]. In the once and twice spawners examined in this study, the peak of V was lower, at approximately 30 mg/mL; however, the general pattern is consistent with that observed in previous data [5, 20]. Following the data reported by Maitra et al. [21] in the Mrigal carp (*Cirrhinus mrigala*), plasmatic levels of this lipophosphoprotein complex are expected to fall to undetectable basal levels after spawning. Moreover, it is worth noting that in this study, comparisons of the plasmatic levels of E2 and V between once and twice spawners reveal a chronological delay in which twice spawners displayed a compressed cycle due to two spawning events in the same year.

 The present findings offer new contributions regarding the reproductive physiology of biannually spawning rainbow trout, further extending characterization of the different attributes of this trait, such as the reproductive performance [6–10] and the underlying genetics [22]. We hope that this work provides more comprehensive knowledge of the oogenesis of twice spawner rainbow trout broodstocks, a strain that has potential to contribute to new high-fecundity broodstocks for farming.
